# A Survey of Gene Prioritization Tools for Mendelian and Complex Human Diseases

**DOI:** 10.1515/jib-2018-0069

**Published:** 2019-09-09

**Authors:** Olga Zolotareva, Maren Kleine

**Affiliations:** Bielefeld University, Faculty of Technology and Center for Biotechnology, International Research Training Group “Computational Methods for the Analysis of the Diversity and Dynamics of Genomes” and Genome Informatics, Universitätsstraße 25, Bielefeld, Germany; Bielefeld University, Faculty of Technology, Bioinformatics/Medical Informatics Department, Universitätsstraße 25, Bielefeld, Germany

**Keywords:** Data integration, Gene prioritization, Human diseases

## Abstract

Modern high-throughput experiments provide us with numerous potential associations between genes and diseases. Experimental validation of all the discovered associations, let alone all the possible interactions between them, is time-consuming and expensive. To facilitate the discovery of causative genes, various approaches for prioritization of genes according to their relevance for a given disease have been developed. In this article, we explain the gene prioritization problem and provide an overview of computational tools for gene prioritization. Among about a hundred of published gene prioritization tools, we select and briefly describe 14 most up-to-date and user-friendly. Also, we discuss the advantages and disadvantages of existing tools, challenges of their validation, and the directions for future research.

## 1 Introduction

Gene prioritization problem emerged together with the growth of popularity of genetic linkage analysis. Genetic mapping yielded large loci containing many candidate genes, only a few of which were indeed associated with the investigated phenotype. In order to determine causative gene variants, dozens of candidate genes from those loci had to be examined and it was economically reasonable to start from genes more likely to impact the observed phenotype. Selection of the most promising candidates can be done on the basis of previous knowledge about these genes, e.g. functional associations or similarity with known disease genes. In the early 2000s, aggregation of publicly available sequence data and growth of functional annotation of the human genome enabled automatization of gene prioritization [1]. In 2006 Aerts et al. [2] prioritized 58 candidate genes from a 2-Mb region of chromosome 22 according to their similarity with known disease genes in ten distinct evidence sources. They predicted YPEL1 as a novel gene involved in atypical DiGeorge syndrome (DGS) and validated this prediction *in vivo*: knock-down of YPEL1 homolog in Zebrafish embryos led to craniofacial defects and confirmed its role in pharyngeal arch morphogenesis [2].

With the advent of high throughput methods, genetic linkage analysis was replaced by Genome-wide association studies (GWAS) [3] allowing cheaper, faster and more precise genetic mapping. However, the necessity of ranking long list of candidate genes according to their relevance to phenotypes had not disappeared. Modern high-throughput experiments, such as genome-wide association studies (GWAS) or differential expression studies generate hundreds or thousands of potential associations, requiring further exploration. Some of these associations may appear by chance or due to systematic biases and therefore may be poorly reproducible. In parallel with the simplification of the candidate gene search, the amount of available information about genes increased. This information includes the data on gene-gene interactions or interactions of genes with other biological entities, their involvement in various biological processes, such as the development of disorders or other phenotypic traits. The emergence of various biological databases and the explosive growth of relevant scientific publications further complicated manual evaluation of candidate genes and stimulated the development of computational methods and tools for gene prioritization. Gene prioritization tools were extensively applied for prediction of genes involved in Mendelian [2], [4], [5], [6] and complex diseases [7], [8], [9], [10] and other polygenic traits [11], [12]. In addition to the evaluation of gene relevance for single diseases, gene prioritization was used for the selection of genes potentially responsible for the comorbidity between two complex diseases – asthma and hypertension [13]. Moreover, taking into account the predicted importance of candidate genes, i.e. score assigned in the result of prioritization, improved the results of pathway enrichment analysis [7], [11], [12], enhanced models for drug response [14], and disease outcome predictions from gene expression profiles [15].

At the moment, hundreds of research papers on gene prioritization have been published [16] and about a hundred of them describe computational tools. In the course of development of this field, many reviews [17], [18], [19], [20], [21], [22], [23] and benchmark [24], [25], [26] works have been published. However, either they cover a very small number of tools and are more focused on theoretical aspects [27] or describe outdated programs no longer supported. Recently, Seyyedrazzagi and Navimipour [16] published a comprehensive literature review where they selected 19 gene prioritization methods for comparison. However, only 7 of those 19 works published between 2011 and 2015 provided links to either web service or code. We managed to access five of them and successfully run only one tool, ProphNet [28], which became unavailable during the preparation of this work. Therefore, in contrast with Seyyedrazzagi and Navimipour [16], we focused on articles describing computational tools and inspected their availability and usability. This review is aimed to give an updated view of existing gene prioritization tools with a specific focus on the most competitive, user-friendly and up-to-date tools. Here, we provide the classification of gene prioritization techniques, develop criteria for selection of the most up-to date and user-friendly tools and briefly describe selected tools.

## 2 Overview of Gene Prioritization Tools

**Figure 1:**
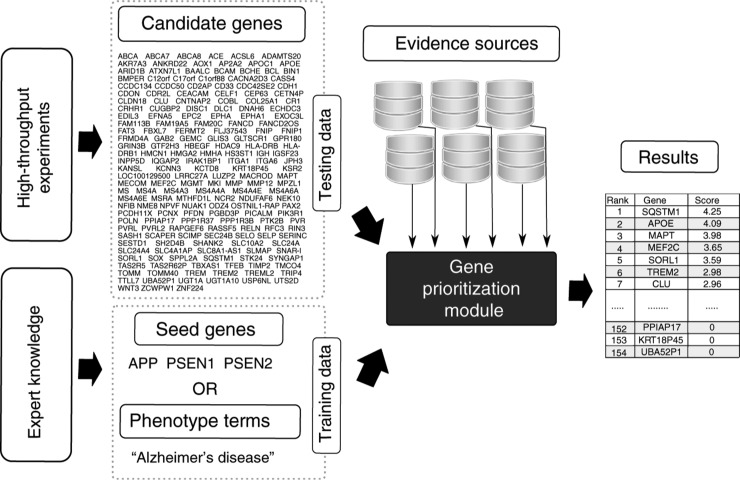
The scheme of a gene prioritization tools. Gene prioritization tools extract information about specified candidates and seed genes or phenotype terms from evidence sources and calculate a score that reflects how likely each gene is responsible for the development of a phenotype. In this example, genes which have alleles causing an early-onset autosomal dominant familial form of Alzheimer’s disease are used as seeds. Candidate genes were obtained from GWAS Catalog [29]. Each candidate gene has at least one variant associated with Alzheimer’s disease. The output of the program is a ranked list of candidate genes arranged according to calculated scores.

Gene prioritization task could be formulated as follows: arrange candidate genes in order of their potential to be truly associated with the disease decreasing on the basis of prior knowledge about these genes and the disease. A typical gene prioritization tool is composed of two parts: a collection of evidence sources (i.e. databases of associations between genes, diseases and other biological entities) and a prioritization module (Figure 1). Prioritization module takes two inputs: training data, which is used to define a phenotype of interest and testing data, a set of user-defined candidate genes to prioritize. After that, it extracts information about given genes or terms from evidence sources and calculates a score that reflects “likelihood” of each gene to be responsible for the phenotype. Training data could be represented either by genes, that were previously linked with a phenotype (*seed genes*). Alternatively to seed genes, some tools (PolySearch2 [30], PhenoRank [31], Open Targets [32] and others [28], [33], [34], [35], [36]) require phenotype or disease terms defining relevant gene-disease associations. The second part of the input is a set of candidate genes to prioritize or in some cases, the whole genome [28], [30], [31], [32], [36], [37], [38]. Some tools, such as Génie [35], Open Targets and Endeavour [39], can automatically construct a set of candidate genes, belonging to a specific class, e.g. all protein-coding genes [35], all receptors or enzymes [32], or use GO or pathway terms to define a functional group of genes [30], [39]. The output of the program is a list of candidate genes arranged according to calculated scores or *p*-values. Every gene prioritization tool represents a unique combination of evidence sources, prioritization strategy and input requirements.

### 2.1 Prioritization Strategies

In previous works, gene prioritization tools have been classified based on the scope of their application (generic or disease-specific) [19], types of evidence sources used [20], approaches (filter-based selection or ranking) [19], [21] and method types (network analysis, similarity profiling or text-mining) [21], [23]. In this review, we propose two classifications of gene prioritization tools: (i) by assumptions they rely on, and (ii) by data repesentay use. It is important to note that the proposed categories are not mutually exclusive, and the purpose of this chapter is to provide the reader with a general idea on the most popular gene prioritization approaches.

#### 2.1.1 Assumptions

The existing gene prioritization approaches rely on two major assumptions. First, genes may be directly associated with a disease, if they are systematically altered in the disease compared to controls (e.g. carry a disease-specific variant). Although various associations may have different strengths and qualities, it is assumed that association, supported by multiple independent studies is more likely to be true. Second, genes can be associated with a disease indirectly, via *guilt-by-association* principle, assuming that the most probable candidates are somehow linked with genes or other biological entities, that were previously shown to impact the phenotype of interest.

Two types of gene prioritization strategies can be distinguished, depending on the assumption they rely on and, consequently, on the kind of prior knowledge used to solve the gene prioritization problem. Strategies of the first type integrate for each candidate all evidence supporting its association with the query disease and compute the overall score. Such tools require a user to provide keywords or ontology terms specifying the disease and then integrate gene-disease associations of various kinds (subsection 2.2.1). In contrast, strategies of the second type reduce the gene prioritization problem to the task of finding genes closely related to known disease genes and, instead of specifying the disease explicitly, accept a set of seed genes, implicitly defining the disease. These tools integrate and analyze associations between genes (subsection 2.2.2), direct and indirect, and prioritize candidates by their similarity and/or proximity to a set of seeds.

Although the majority of tools follow exclusively one of these two strategies, some tools implement a combination of them. For example, PhenoRank [31] and Phenolyzer [33] accept disease keywords, automatically construct a scored list of seed genes, and rank the rest of genes such that genes associated with high-scored seeds also get higher ranks. Another example is NetworkPrioritizer, which retrieves genes associated with a query disease, builds disease-specific network and identifies the most relevant genes based on the network topology [40].

#### 2.1.2 Data Representation Models and Prioritization Approaches

**Figure 2:**
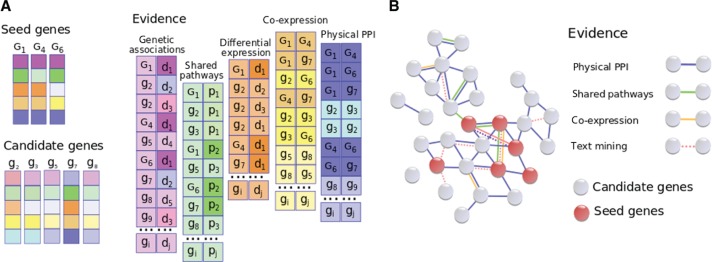
Data representation models utilized by gene prioritization tools. A. Relational data structure. The first and the third evidence sources provide relationships between genes labeled with *G* (seeds) or *g* (candidates) and diseases (*d*), the second source provides gene membership in pathways (*p*) and the last two evidence sources contain different kinds of interactions between genes. Vector representation of seed and candidate genes are shown on the left. The similarity between colorings of gene *g*_7_ and seed genes shows that *g*_7_ seems to be a promising candidate. B. Network data structure. Nodes depict genes, edges show relationships between genes. Seed genes are highlighted with red. Types of interactions and associations are shown on the right.

The structure of evidence sources utilized by a gene prioritization tool can be either relational (Figure 2A), when data sources are represented by a collection of tables, containing an association of a particular kind, or network (Figure 2B), where nodes correspond to genes (or other entities) and edges represent relationships between them. Although these two data representations models are interchangeable, organization of evidence sources is always consistent with the prioritization algorithm. Accordingly, most of the existing approaches can be classified as score aggregation or network analysis methods, or represent their combination [41], [42]. Since the proposed classification is very general, we discuss the methods of network analysis and score aggregation below.

##### Network Analysis

Network is a natural representation of pairwise entity relationships, widely used to describe similarities or interactions between biological objects. Many independent studies agree that disease-associated proteins tend to cluster on the network of protein-protein interactions [43], [44], [45] (PPI). This observation became the basis of many gene prioritization approaches assuming that proteins, encoded by unknown disease genes and more tightly connected with known disease proteins than irrelevant proteins. Moreover, disease proteins were shown to possess special network properties, for example, they tend to be more central in the disease-specific networks [46], [47]. This observation is used in some network-based gene prioritization workflows [40], [46], [48], [49] including also those omitting seed genes [13], [15]. Briefly, these methods retrieve all genes somehow associated with a query disease, build disease-specific interaction network and determine the essentiality of all nodes considering the network topology.

The majority of network-based tools require seed genes and rank candidate according to their proximity in the network toseeds. The distance from a node to a group of nodes in the network may be defined in numerous ways. MaxLink [50], [51] ranks first neighbours of known genes according to a number of direct links to them. In order to reduce hub bias, it takes into account only candidates which have significantly more connection with seed genes than expected by chance. Similarly with MaxLink, DIAMOnD [38] applies hypergeometric test to detect candidates enreached by seed genes among their first neighbours. In contrast, DIAMOnD ranks genes according to significance of seed overrepresentation among the first neighbours. In every iteration, DIAMOnD includes the most significant candidate into a set of seeds and recalculates *p*-values for the remaining candidates regarding the updated set of seeds. However, MaxLink considers only first neighbours of seeds and DIAMOnD ignores indirect interactions on every iteration. Gentrepid [52] ranks candidates conforming to the shortest path distance to a seed node. NetShort method implemented in GUILD framework [53] down-weights edges connected to genes with a high score when computing shortest path length. The disadvantage of this measure it that not all the pathways are equally informative, e.g. a path going through promiscuous hub nodes may be short but unspecific to the disease mechanism. In order to address this problem, various network propagation methods, modeling information flow over the network have been developed. ToppNet [54], GeneWanderer [55], PhenoRank [31] and many others [56], [57], [58], [59], [60], [61], [62] apply random walk-based algorithms [63], [64], [65] in order to assess relative importance of a node to a group nodes considering the global network topology. Other methods mathematically related [66] with random walk, modelling diffusion [67], [68], [69] or electric current flow [70] through the network have been used successfully in gene prioritization. GeneMANIA [71] implements Gaussian field label propagation algorithm [72], which redistributes seed gene scores to their neighbors, minimizing differences between both scores of neighboring genes and initial and assigned scores of seed genes. PRINCE [67] uses conceptually similar approach to smooths influence of disease genes over the network. It simulates the exchange of flows between genes in the network, where every node produces outcoming flows to neighbors, proportional to its score, and computes a new score summarizing incoming flows. The process starts from disease genes and stops after many iterations. In the result, candidates connected with many disease genes gain higher income flow and thus a higher score. eQED [70] represents the network as an electric circuit where seeds are current sources, edges are conductances, candidates are drains, and rank candidates by current flowing through them. Köhler et al. [55], Navlakha and Kingsford [73], and Shim et al. [25] have shown that methods considering global network topology demonstrate higher overall performance than methods based only on local network information. At the same time, methods using local network topology, e.g. direct interactions or shortest path distances, rank true top-ranked candidates higher [25], [26] and therefore are more successful for diseases with few associated genes, tightly connected in the network [25].

Another important feature determining the performance of the network analysis tool is the network type used, its quality and completeness [53]. Some network-based prioritization tools use homogeneous networks modeling only one type of interactions [30], [36], [54], [74]. However, recent studies demonstrate that composite networks, composed of many various kinds of interactions and relationships, outperform any single network, possibly because individual networks contain complementary information [75], [76]. Therefore, many gene prioritization tools use functional protein interaction networks such as GeneMania [37], FunCoup [77], STRING [78] or integrate several networks of different types [33], [38]. Moreover, in previous works gene prioritization is performed on heterogeneous networks including multiple types of biological entities [28], [56], [61], [62], [79], [80].

##### Score Aggregation

This group includes tools implementing various strategies of aggregating all found pieces of evidence within each data source and then between different data sources into a total score. For example, Polysearch [30], [74] recognizes sentences supporting gene-disease associations, weights them according to their reliability and summarizes weights into the total relevancy score. When relevancy scores computed for all genes, Polysearch standardizes them and uses for prioritization. Similarly, DisGeNET [34], [81], [82] and Open Targets [32] integrate data from multiple evidence sources. For each gene, they compute a weighted sum over all individual gene-disease association scores. Each weighting coefficient is determined by the reliability of association and the type of data source it came from. Thus, strong genetic associations discovered in human make a bigger impact into the overall gene score, than less reliable associations inferred from animal models or text mining.

Tools operating with seed genes employ similar idea to summarize gene-gene associations. Initially, they score each candidate by its similarity with seeds, considering each evidence sources independently, and then combine all data source-specific scores into a total score. GPS [42] follows the most straightforward way to integrate multiple rankings: for each gene, it calculates a simple rank average over seven independent rankings. ToppGene [54], [83] and Endeavour [2], [39], [84] realize more sophisticated approaches to obtain the overall ranking. They convert data source-specific scores into *p*-values and apply meta-analysis-based techniques to compute the overall *p*-value for each gene.

Score aggregation approaches described above have at least two drawbacks. First, these tools favor genes top-ranked in a maximal number of evidence sources. Meanwhile, they may not consider various reliability and potential dependency of evidence sources. Second, tools from this category do not take into account the fact that the impact of independent rankings into the total score may not be additive.

These deficiencies have been partly overcome with the development of machine learning methods. Similar to ToppGene and Endeavour, machine learning-based methods represent genes as *n*-dimensional feature vectors, use seed genes as positive training exemplars, genes other than seeds or candidates as negative exemplars, and then classify candidates. Machine learning methods such as multiple linear [41], [85], [86] and logistic [33], [87] regressions, kernel-based approaches [88], [89], [90], neural networks [91] and others [92] were successfully applied for gene prioritization. Recent works have demonstrated that machine learning-based methods tend to outperform other score aggregators [87], [93], [94], possibly owing to their ability to capture unknown or non-linear feature relationships and tuning model parameters.

### 2.2 Evidence Sources

Almost all gene prioritization tools with rare exceptions [95], [96] rely on internal databases integrating a wide variety of information sources. In this paragraph, we discuss types of evidence used for establishing of gene-disease associations and for calculation of gene similarities. Performance of gene prioritization tools strongly depends on variety data sources used [2], [18], [97], [98] and on their novelty [24].

#### 2.2.1 Gene-Disease Associations

##### Genetic Associations

Since many of human diseases are proven to have a genetic component, the discovery of genetic variants underlying diseases is one of the major challenges of human genetics. Impact of the individual genetic constitution on the development of the disease may be very different: from highly heritable Mendelian disorders, e.g. sickle-cell anemia (OMIM:#603903) or Duchenne muscular dystrophy (OMIM:#310200) caused by mutations in certain genes, to infectious diseases [99] caused by an external pathogenic agent. Several thousands of the human diseases are caused by the dysregulation of a single gene: loss [100] or modification [101] of its function. These diseases are usually rare and aggregate in families which carry a specific mutation. In addition to medical significance, such monogenic diseases allow connecting phenotypes and genes and thus providing us with clues about gene functions. Therefore, one of the main and widely used sources of knowledge for gene prioritization is OMIM [102]. OMIM provides a constantly updating catalog of hereditary disorders and associated genes. Although OMIM initiated as a database of Mendelian diseases, now in addition to more than 5000 single-gene traits, it coves about 700 complex and 150 non-disease phenotypes. Besides OMIM, several other databases collect information about rare monogenic diseases and disease-causing variants: OrphaNet [103], DECIPHER [104], ClinVar [105], HGMD [106]. In addition to comprehensive catalogs of human monogenic diseases, one can obtain genotype and phenotype information on animal models of human diseases from species-specific databases e.g. Mouse Genome Database [107] and Rat Genome Database [108].

Unlike Mendelian diseases, complex diseases cannot be explained by a single mutation with high effect but thought to be the result of interactions between multiple genetic and environmental factors. Genome-wide association studies (GWAS) discover common single nucleotide polymorphisms (SNPs), which allelic states significantly correlate with disease status. NHGRI-EBI GWAS Catalog [29] provides a curated and regularly updated lists of published GWAS, and contains over 60,000 of SNP-trait associations from 3411 publications. However, effects of such risk variants identified in GWAS are much weaker than effects of Mendelian variants. Moreover, linking a variant with its effect on a certain gene may be a challenging task because, in contrast with Mendelian disease variants, only a small fraction of GWAS hits has an obvious effect on the protein, e.g. missense substitution or frameshift. Most of significant GWAS variants locate in intronic or intergenic regions [109]. Mapping them to the closest gene may not always be correct, and considering regulatory annotations for mapping of silent GWAS variants to genes appears to be a better strategy [110]. Joehanes et al. [111] have shown, that about half of GWAS variants lay in expression quantitative trait loci (eQTL) – genome regions with markers correlated with expression levels of one or several genes.

##### Differential Expression

Besides DNA sequence variations, many other kinds of biological evidence may be used for the inference of gene-disease associations. Since disease manifestation usually accompanied by various molecular changes, case-control omics studies allow identifying coding and non-coding transcripts [112], proteins [113], and other entities such as metabolites [114] and epigenetic marks [115] altered in disease samples compared to controls and thus potentially involved into the pathogenesis. Genes and proteins differentially expressed under various biological conditions, including diseases and developmental stages in human and other organisms can be obtained from Expression Atlas [116], a curated database of expression profiles in human and many other species derived from selected RNA-seq and microarray datasets. Expression Atlas uniformly processes and analyse expression data obtained from multiple sources, from individual Gene Expression Omnibus [117] datasets to large expression studies, such as GTEx [118] and Human Protein Atlas [119], [120]. Also, Expression Atlas provides baseline expression levels in tissues and cell types and allows retrieving tissue- and cell type-specific genes. This information can also give a hint about gene function. For example, geneTIER [121] assumes that plausible candidate genes are highly expressed in tissues, affected by the disease. However, this assumption is only applicable when affected tissues or cell types are well known, which is not always the case.

##### Other Ways of Establishing Gene-Disease Associations

In addition to gene-disease associations identified in case-control experiments, some indirect associations can be inferred through a third biological entity, utilizing *guilt-by-association* principle. The next paragraph discusses indirect gene-disease associations mediated by known diseases genes in deep details. Moreover, indirect gene-disease associations can be mediated by biological entities other than genes: chemicals [122], tissues [79], and other diseases and phenotypes. For example, a gene targeted by a drug used to treat the disease, it is likely to participate in the mechanism of the disease. The same may be true for targets of drugs and toxic compounds are known to cause negative effects e.g. adverse reactions, similar to the disease. Such gene-disease associations inferred from curated pairwise associations with chemicals, which can be obtained, for example, from Comparative Toxicogenomic Database [122] (CTD, http://ctdbase.org/). Similarly, putative gene-disease associations can be established through the second disease, via disease correlation [123] or symptom similarities [124]. Since co-occurring diseases tend to have more shared genes, than expected by chance [123], at least some of genes, known to be associated with one disease may be involved into the other disease. The same is true for diseases demonstrating phenotype similarities [124]. Each human disease can be characterized by a specific combination of multiple phenotypes and, in turn, some phenotypic abnormalities can manifest in many diseases. Thus, brachydactyly syndrome (OMIM:#112410), characterized by age-dependent hypertension, shortening of both phalanges and many other abnormalities [101]. At the same time, hypertension is a symptom of many other disorders, e.g. #613677, #614495, #500005, #218030, #602531. In order to annotate diseases with phenotypes and calculate phenotypic similarities between diseases, unified ontologies for diseases [125] and phenotypes [126], [127], [128] were developed. Human Phenotype Ontology (HPO) project also provides the results of semi-automatic mapping between phenotypes, disease, and genes [127]. Recent the Monarch Initiative [129] allows comparing human phenotypes with animal phenotypes [130] with a known genetic basis.

#### 2.2.2 Associations between Genes

##### Physical Interactions

Gene prioritization approaches are based on *guilt-by-association* principle and assume that the most promising candidates are in some way associated with seed genes. Physical PPI point to potential functional interaction between these proteins and subsequently, to the association between corresponding genes. Physical PPI can be experimentally identified using high-throughput methods, such as yeast two-hybrid assay, affinity purification with mass spectrometry or confirmed in single experiments, e.g. X-ray crystallography. Primary PPI databases obtain data from curation of published literature, e.g. DIP [131], HPRD [132], BioGRID [133], InnateDB [134] or MatrixDB [135] or from single large-scale experiments [136], [137]. Other PPI databases, such as IntAct [138], MINT [139], MENTHA [140], HitPredict [141], integrate protein interaction data from multiple primary databases and assign interaction reliability scores according to the supporting evidence. In order to facilitate an access to a large number of redundant PPI databases, a standardized query interface PSIQUIC was created.

In addition to direct physical contacts, proteins can also interact indirectly, collectively performing their function. For example, since a protein complex functions as a whole, all its members, including those non-interacting directly, are strongly functionally related. CORUM [142] and Complex Portal [143] provide curated human and animal protein complexes, their subunit composition, structure and functions.

##### Pathways and Regulation

Proteins participating in consequent steps of a biological pathway are also considered to be functionally related. In a broad sense, biological pathway is a chain of molecular events, such as chemical reactions, conformational changes, binding or dissociation, etc., which leads to certain changes in the cell. Pathguide [144] is a comprehensive catalog comprising of 702 resources related to pathways and molecular interactions in human and other organisms. Pathways are classified according to prevailing interaction type as metabolic, signaling, and regulatory. Metabolic pathways, representing chains of chemical reactions catalyzed by enzymes, can be found in MetaCyc [145], which is a part of BioCyc, including pathway-related information for more than 13,000 species. Signalling databases, such as OmniPath [146], Signor [147], SignaLink [148], PhosphoSite [149], contain literature-curated information on cellular signal transduction via post-translational modifications, relocation, binding or conformational changes. Genetic regulation databases contain manually curated and computationally inferred relationships between genes and transcriptional factors (TFs), e.g. JASPAR [150], TRANSFAC [151], or miRNA, e.g. miRTarBase [152]. Large pathway databases, such as KEGG [153], Reactome [154] and ConsensusPathDB [155] are not specialized on a particular type of pathway or process and provide biological interaction of multiple types for human and other organisms, while the other resources have a certain focus, e.g. innate immunity [134] or a specific disease [156], [157].

##### Predicted Interactions

Since biological pathways are mediated by gene products, proteins or RNAs, pathway data is the invaluable source of functional relationships between genes. However, known pathways cover only a small part of all the existing interactions and not all human genes are fully functionally annotated. Unknown gene functions and interactions can be computationally predicted on the basis of gene co-expression [158], sequence similarity [159] or interactions [160], [161] with well-annotated genes. Genes or proteins with expression level correlated across different conditions are likely to be co-regulated and may share functions [158]. Sequence similarity and domain composition can also give a clue about a function of an unannotated protein and help to identify its interaction partners. Recent paralogs may have the same function [162], but later their functions tend to diverge. Orthologs are more functionally conservative [163] and therefore functional annotations of genes from related species and PPI [164] may be transferred on their human orthologs.

The amount of knowledge regarding gene and protein roles in the cell is diverse, enormous and continuously growing. The unification and formalization of this knowledge are crucial to ensure its computational processing and analysis. Gene Ontology consortium [165] created in 1999, develops and maintains a controlled vocabulary of concepts describing gene functions, localizations and participation in biological processes. GO consortium provides regularly updating [166] whole-genome annotations, either supported by experimental evidence or computationally predicted, for multiple species, from human to bacteria, which allows within and between-species comparisons of gene functions. GO term enrichment analysis became a community standard for functional annotation of gene sets and interpretation of the experiment results. Since genes sharing GO terms are considered to be functionally related, many gene prioritization tools utilize GO as an additional source of evidence.

#### 2.2.3 Text Mining

Yet another way of establishing putative associations between genes, diseases and other biological entities is text mining of biomedical literature. Many gene prioritization tools utilize the results of co-occurrence based text mining, assuming that frequent colocalization of two entities in biomedical texts points to their possible interaction. More sophisticated pattern-based text-mining methods use advanced weighting schemes to assign qualities to predicted associations [30], [74]. Other text-mining systems, e.g. ANDsystem [167], apply natural language processing (NLP) algorithms allowing to differentiate between various kinds of biological entities and associations between them.

Some gene prioritization tools consider text mining-inferred associations together with curated and predicted associations from other evidence sources when some of them (e.g. Génie [35], GLAD4U [36]) completely rely on the text-mining results. The major drawbacks of text mining methods are the high rate of false positives and the lack of accuracy in the detection of associations and determination of their types. Despite that, text mining remains the only way to absorb the whole volume of relevant scientific literature, impossible to handle manually.

### 2.3 Selection of Related Works

Since many of research papers on gene prioritization describe the method but provide no code or link to the tool, in order to achieve better coverage of existing tools we were not confined to works found in Pubmed and Google Scholar by keywords “gene”, “prioritization” or “prioritizing” and “disease”. In addition, we searched in specialized software catalogs Gene Prioritization Portal (http://homes.esat.kuleuven.be/~bioiuser/gpp/) and OMICtools (https://omictools.com/) and included in the list all found tools which were published in scientific journals. Aside from tools, explicitly classified as gene prioritization tools by authors, we included into this review several multi-purpose tools, such as Polysearch [30], [74], able to prioritize associations between various biological entities, including, but not limited to genes and diseases. Also, some large biological data portals such as DisGeNET [34], [81], [82], Open Targets [32] and GeneMania [37], [71], [168] providing engines for gene prioritization, although it is not their main purpose, were added to comparison. We did not take into account computational tools or resources which can be used for gene prioritization, but required implementation of essential parts of gene prioritization workflow, e.g. scoring functions [79], [169]. Also, it is important to note that tools developed to solve related, but different problems such as variant prioritization [170], [171], [172], prediction of protein function [173] or detection of cancer driver genes [174] were left out of the scope of this review. We also did not consider gene prioritization tools developed specifically for non-human organisms [175], [176], or working with a very limited set of human phenotypes, e.g. only for neurodegenerative diseases [177] or cancers [178]. Despite such tools often use data more relevant for a specific task, they might be less interesting for a broader audience than generic gene prioritization tools. In total we have found references to 96 published tools for gene prioritization in human disorders (Table S1).

### 2.4 Selection and Characterization of the most Promising Tools

**Figure 3:**
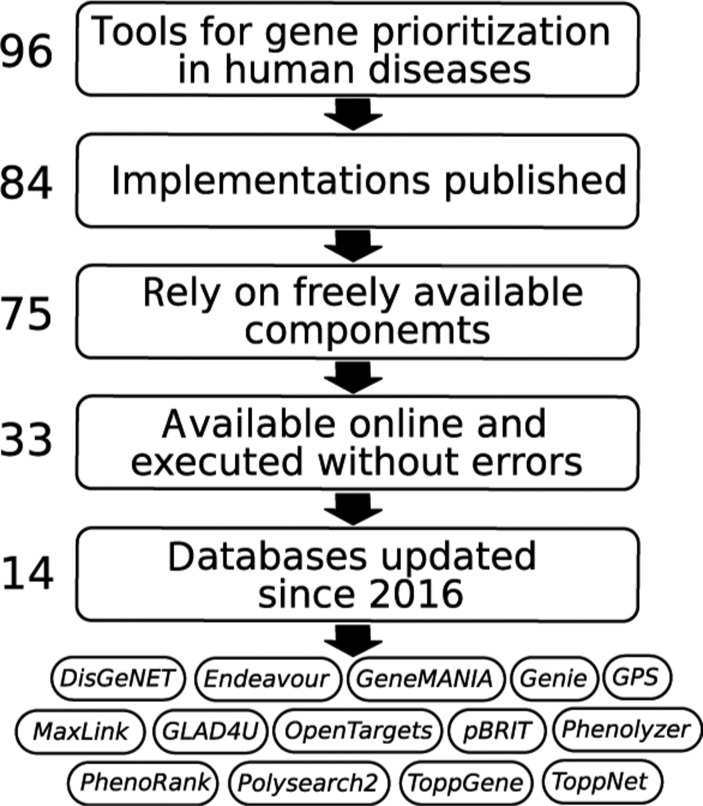
The process of gene prioritization tools selection for further detailed comparison.

Since many of gene prioritization tools were not available or not supported by their authors, we decided to focus on the most promising ones. The process of selection of the most up-to-date and user-friendly tools for gene prioritization in human disorders is shown in Figure 3. First of all, we excluded from consideration methods which implementations were not published (i.e. no web link was found), what reduced the list to 84 tools. Next, we kept only open source tools with implementations relying on freely available components and tried to run the remaining 75 tools. After exclusion of tools which were offline, failed to run or executed with errors, the list reduced down to 33 tools. Thus, only 34% of 96 gene prioritization tools published since 2002 remained available for users at the beginning of 2018. Finally, we favored tools with the most up-to-date evidence sources, because the completeness of evidence sources is crucial for gene prioritization tool performance [24]. Therefore we checked the dates of the last database update and selected for detailed comparison 14 tools, which have updated their databases since 2016.

We categorized these 14 gene prioritization tools according to assumption and prioritization approach they use (Table 1) and types of evidence sources they rely on (Table 2). Five tools integrated gene-disease associations and accepted disease terms instead of seed genes. Seven other tools searched for genes associated with user-defined seeds and four of them applied network analysis techniques. Two remaining tools, Phenolyzer and PhenoRank, took into account both gene-gene and gene-disease associations and automatically identified most relevant genes for given diseases terms. Brief descriptions of the methods used by all 14 tools and their additional parameters are provided in Supplementary Table 2.

We also paid attention to interfaces of selected tools because they correspond to the level of expertise required from the user. All of the selected tools, except PhenoRank [31], had web interfaces and therefore were available for a broad audience of users without special programming skills. However, only 6 tools provided programmatic access options (e.g. web API interfaces, scripts or command line tools), advantageous for tasks requiring batch execution.

**Table 1:** The characterization of selected gene prioritization tools.

	strategy		approach type		interfaces		input		
	integrate gene-disease associations	search for genes associated with seeds	score aggregation	network analysis	web interface	programmatic access	seed genes	disease or phenotype terms	candidate genes
**DisGeNET [34]**	+		+		+	+	no	yes	whole genome
**GLAD4U [36]**	+		+		+		no	yes	whole genome
**Genie [35]**	+		+		+		no	yes	optional
**OpenTargets [32]**	+		+		+	+	no	yes	optional
**Polysearch [30]**	+		+		+		no	yes	whole genome
**Phenolyzer [33]**	+	+	+		+	+	no	yes	optional
**PhenoRank [31]**	+	+		+		+	no	yes	whole genome
**Endeavour [39]**		+	+		+		yes	no	yes
**GeneMANIA [37]**		+		+	+	+	yes	no	whole genome
**GPS [42]**		+	+	+	+		yes	no	yes
**MaxLink [51]**		+		+	+		yes	no	whole genome
**pBRIT [85]**		+	+		+	+	yes	no	yes
**ToppGene [83]**		+	+		+		yes	no	yes
**ToppNet [74]**		+		+	+		yes	no	yes

**Table 2:** Types of evidence sources used by each of 14 gene prioritization tools.

	Gene Interactions					Gene Similarities									Gene-Disease associations						
	Physical PPI	Pathways	Genetic interactions	Regulation	Interologs	Co-expression	Co-localization	Functional annotations	Phenotype similarity	Shared domains	Sequence similarity	Phylogenetic profile similarity	Chemical interaction	Text Mining	Genetic associations	Differential expression	Animal models	Human phenotype similarity	Chemical information	Pathways	Text mining
**DisGenNET**															**+**		**+**	**+**	**+**		**+**
**GLAD4U**																					**+**
**Genie**																	**+**				**+**
**OpenTargets**															**+**	**+**	**+**		**+**	**+**	**+**
**Polysearch2**																					**+**
**Phenolyzer**	**+**	**+**		**+**				**+**			**+**				**+**						
**PhenoRank**	**+**								**+**						**+**		**+**				
**Endeavour**	**+**	**+**	**+**	**+**	**+**	**+**	**+**	**+**	**+**	**+**	**+**		**+**	**+**							
**GeneMANIA**	**+**	**+**	**+**		**+**	**+**	**+**		**+**	**+**			**+**								
**GPS**	**+**	**+**	**+**			**+**		**+**		**+**	**+**	**+**									
**MaxLink**	**+**		**+**	**+**	**+**	**+**	**+**					**+**									
**pBRIT**	**+**	**+**						**+**	**+**		**+**			**+**							
**ToppGene**	**+**	**+**		**+**		**+**		**+**	**+**	**+**	**+**		**+**	**+**							
**ToppNet**	**+**																				

## 3 Evaluation of Gene Prioritization Tools

Although some individual tools may demonstrate superior performance on average, none of them outperform in all cases [24], [25], [26], [179]. This may happen because of the different capability of methods to model aspects relevant to the disease mechanism, or due to the usage of distinct validation approaches and different training and testing datasets. In this chapter, we discuss approaches for validation of gene prioritization results, measures of performance, and their advantages and disadvantages.

### 3.1 Validation Data

Despite gene prioritization results in a ranked list of genes, in many works, it is turned into a binary classification task, where genes associated with the disease must be distinguished from unrelated ones. The assessment of classification quality requires multiple positive and negative instances, i.e. genes associated and not associated with a disease. Many studies prove the ability of gene prioritization tools to facilitate the identification of genes carrying monogenic diseases variants, e.g. [2], [180]. Gene prioritization can also advance identification genes relevant for complex traits, allow selection the most promising candidates for GWAS [181] and selection of targets for RNA interference screening [182]. However, none of these studies represents performance evaluation in its strict sense, with the determination of type I and type II error rates and experimental verification of gene roles.

Since experimental testing of a sufficiently large number of genes is expensive and time-consuming, most of the researchers turn to publicly available databases or scientific papers and construct golden datasets of high-confidence curated associations [24], [33]. Genes without any evidence of association with the disease are considered as negative exemplars. Finally, positive and negative instances are distributed between testing and training sets (if necessary) and are used to calculate one or several performance measures.

### 3.2 Measures of Performance

Although many evaluations of gene prioritization tools are described in literature [24], [25], [26], no unified approach for evaluation of gene prioritization tool exists. Doncheva et al. [20], and Gill et al. [23] provide a good overview of performance measures applicable for evaluation of gene prioritization tools. Optimal validation approach and measure of performance depend on the nature of the disease (monogenic or polygenic) and tolerance to false positives. For monogenic or oligogenic [183] diseases by definition just one or few causative genes exist and their experimental validation is time-consuming and laborious. Therefore, in the case when only a few candidates can be tested, suitable performance measures are the averaged number of false candidates appeared above the right one, or success rate, when the correctly identified disease gene was ranked within *n* top genes.

In contrast with monogenic diseases, complex disorders are associated with multiple genes, and the expected number of true associations may vary depending on the disease. Although the mean rank ratio of true positive findings is applicable in this case, it provides little information about the overall distribution of the true positive ranks. For complex disorders, potentially associated genes are not necessarily tested individually but may be analyzed as a group. For some tasks, such as gene set or pathway enrichment analysis, individual gene ranks are not so crucial as the enrichment of true positives in the top of the list. Averaged fold-enrichment [20], [41] shows the increase of true positive rate (TPR) in the top of the ranked list compared to the background TPR. The method achieves *n*/*m*-fold enrichment on average, if in *n*% of cases correctly identified disease genes are ranked among the top *m*% of all candidates [41]. Besides the TPR, also known as sensitivity, another essential characteristic of performance is specificity, which shows the ability of the method to correctly classify negative exemplars (true negative rate). The relationship between sensitivity and false positive rate (FPR) plotted at variable threshold levels gives a receiver operating characteristic (ROC) curve [184]. The cross-validated area under the ROC curve (AUC) [185] provides an estimate of overall tool performance [26], [31], [39], [85].

### 3.3 Challenges of Validation

Many methods reduce gene prioritization problem to the problem of finding genes most similar to seed genes and thus the result may strongly depend on seed gene selection. Nevertheless, no universal rule on seed selection exists, and the choice of seeds is usually made subjectively. Expert decisions on optimal number of seed genes vary from several to dozens [18], [21], [24] or even over a hundred [186]. However, since seed genes must have a proven role in the disease, for monogenic and oligogenic disorders only one or few such genes exist. Consequently, for these diseases, the methods accepting disease terms and analyzing gene-disease associations bypassing seed genes, e.g. via phenotype similarities are more suitable. In turn, multifactorial disorders have many associations of various confidence and power, but by no means all are causal. Furthermore, none of the multifactorial disorders are fully explained, therefore gold standard datasets may be incomplete or contain false associations and therefore give uncertain performance estimates. Finally, if no evidence of association between a gene and a complex disease found, we still cannot be sure whether this gene is a true negative indeed, due to the incompleteness of current knowledge.

At the same time with a lack of reliable ground truth, there are two issues related to the oversupply of biomedical knowledge and its rapid and non-uniform growth. The first problem also referred to as knowledge bias [2], [55] is that well-characterized genes have a better chance to be ranked higher than unannotated ones, only because they have more connections with other biological entities. This effect can be at least partially eliminated by including large-scale experimental data [19] e.g. whole interactome, co-expression network or the results of GWAS. Cornish et al. [31] suggest an elegant way to reduce the effect of knowledge bias on gene ranking. They developed PhenoRank, which computes empirical *p*-values for each gene score, comparing scores obtained on the real disease with scores for the same genes, prioritized for simulated sets of phenotype terms. This improvement allowed PhenoRank outperforming three other gene prioritization tools [31].

Another problem concerning the reliability of benchmark results is a possible uncontrolled inclusion of testing associations into evidence sources, leading to overestimation of the actual performance of the tool. Tranchevent et al. [39] proposed to adopt the methodology [173] termed time-stamped benchmark, in order to reduce this “knowledge contamination”. They saved novel gene-disease association predictions made at the beginning of 2013 and checked which of them were published during the next 2 years. Although in some works researchers apply time-stamped benchmark [39], [85], while reducing “knowledge contamination”, it excludes from the validation cohort the most reliable and well-studied associations, which may also result in a biased performance estimate.

## 4 Future Directions

With the advance of high-throughput technologies, in the last two decades the volume of biomedical knowledge, the variety of evidence sources and their completeness have constantly increased. Large comprehensive data portals collecting gene and gene-disease associations for human and other organisms have been created. Incorporation of new or rarely used evidence sources and improvement of data quality are important directions for future work in the field of gene prioritization.

Simultaneously with extensive development of evidence sources, great progress in the development of gene prioritization algorithms has been made. Although recent studies have shown that machine learning methods tend to outperform simpler score aggregation methods based on statistical approaches [87], [93], [94], [187], no single method outperforming others exist and different tools seem to be complementary. Therefore, the analysis of the results coming from several tools relying on different principles and data sources remains beneficial.

Besides that, there is also an advancement in usability and flexibility of gene prioritization tools. Modern gene prioritization tools allow selection of the algorithm and tuning its parameters, choosing evidence sources and incorporation of user-defined data. They provide a variety of user interfaces and programmatic access options facilitating integration with other computational tools.

Despite the active development and great success of gene prioritization tools during past decades, some challenges remain not fully addressed. In many works, gene prioritization is referred as the task of ranking genes by their relevance to the disease [17], [20], [21], [23], or, more specifically, according to the probability to be causal for this disease [18], [19], [22], [188]. If for monogenic diseases, one or several validated causal variants exist, complex disorders are associated with some variants, whose causal roles are not always confirmed. Furthermore, in complex disorders, many genes may act together and modify the effect of each other jointly contributing to disease development. Despite this, most of the reviewed tools rank candidate genes separately, what fits Mendelian diseases but for complex disorders may be disadvantageous. Although in some tools [31], [37] scores of candidate genes influence each other, nearly none of them explicitly boosts scores of candidates forming a putative pathway or complex. Therefore, it might be reasonable to separate the task of gene prioritization for complex disorders from gene prioritization for monogenic disorders and reformulate it. Instead of ranking all the candidates independently, one can look for subsets of functionally interconnected candidate genes, enriched by associations with a specified disease [98], [189]. The development of specialized protocols for validation of the results gene prioritization for complex diseases is also necessary.

Another aspect of the problem was not addressed by current gene prioritization tools is disease heterogeneity. Disease heterogeneity implies that the disease may be represented by latent subclasses, phenotypically similar but molecularly distinct. The evidence of heterogeneity are shown for many human diseases, complex and monogenic [190], [191]. In order to take into account disease heterogeneity, one should perform gene prioritization together with analysis of patient-level experimental data. One example of such data integration is variant prioritization, which identifies likely disease-causing rare point mutations [170], [171], [172].

## 5 Conclusions

Among about a hundred of gene prioritization tools published to date, we selected and described 14 most promising ones on the basis of their availability, usability, and novelty. In this review, we classified gene prioritization tools according to underlying assumptions, methodology, and data representation models. An optimal tool for gene prioritization must be chosen considering the specificity of a particular task, namely error tolerance, type of inheritance and availability of knowledge about genes and disease of interest. Also, novelty and type of evidence sources utilized by the gene prioritization tool should be taken into account. Finally, we highlighted the limitations of existing gene prioritization tools and discussed the directions of future research.
